# Comparative Outcomes of Fractional Flow Reserve and Intravascular Ultrasound Guidance for Percutaneous Coronary Intervention in Intermediate Lesions: A Systematic Review and Meta‐Analysis

**DOI:** 10.1155/cdr/6570642

**Published:** 2026-03-27

**Authors:** Eyad Jamileh, Zuha Akhtar, Kanita Farooq, Maria Babu, Mohamed Xamza, Ibrahim Antoun

**Affiliations:** ^1^ Royal Blackburn Teaching Hospital, East Lancashire Hospitals Trust, Blackburn, England, UK; ^2^ School of Medicine, University of Oxford, Oxfordshire, England, UK, ox.ac.uk; ^3^ Kings College Hospital NHS Foundation Trust, London, England, UK, nhs.uk; ^4^ Royal Preston Hospital, Lancashire Hospitals NHS Trust, Preston, England, UK, nhs.uk; ^5^ Barking, Havering and Redbridge University Hospitals NHS Trust, London, England, UK, bhrhospitals.nhs.uk; ^6^ Department of Cardiology, Glenfield Hospital, University Hospitals of Leicester NHS Trust, Leicester, UK, nhs.uk; ^7^ Department of Cardiovascular Sciences, Clinical Science Wing, Glenfield Hospital, University of Leicester, Leicester, UK, le.ac.uk

**Keywords:** coronary artery disease, fractional flow reserve, intravascular ultrasound, major adverse cardiovascular events, percutaneous coronary intervention

## Abstract

**Objective:**

Advanced techniques beyond angiography increasingly guide percutaneous coronary intervention (PCI). Fractional flow reserve (FFR) and intravascular ultrasound (IVUS) are widely used for physiological and anatomical guidance, respectively. This study compares clinical outcomes of FFR versus IVUS‐guided PCI.

**Methods:**

We conducted a systematic review and meta‐analysis that identified studies comparing FFR‐ and IVUS‐guided PCI. Primary outcomes included major adverse cardiovascular events (MACE), all‐cause mortality, cardiac death, nonfatal myocardial infarction (MI) and target vessel revascularisation (TVR). A secondary outcome was the number of PCI procedures performed. Random effects modelling was used for all outcomes.

**Results:**

Six studies comprising 5040 patients (FFR: 2517; IVUS: 2523) were included. There were no significant differences between FFR‐ and IVUS‐guided PCI in the incidence of MACE (RR = 1.13, 95% CI 0.89–1.44), all‐cause mortality (RR = 0.82, 95% CI 0.41–1.64), cardiac death (RR = 1.05, 95% CI 0.60–1.85), nonfatal MI (RR = 1.35, 95% CI 0.72–2.52) or TVR (RR = 1.21, 95% CI 0.81–1.81). Subgroup analyses by study design (observational studies and randomised control trials) showed no significant effect modification for any outcome. There was no significant difference in the number of PCI procedures performed (RR = 0.78, 95% CI 0.59–1.02), although heterogeneity was high (*I* ^2^ = 95*%*).

**Conclusion:**

FFR and IVUS are both effective for guiding PCI, with similar outcomes in terms of MACE, mortality, MI, TVR and PCI procedures performed. Modality selection should be tailored to lesion complexity, institutional expertise and resource availability.

## 1. Introduction

Coronary artery disease (CAD) remains the leading cause of global mortality [1]. Percutaneous coronary intervention (PCI) plays a fundamental role in the management of CAD, which is aimed at restoring adequate perfusion to the myocardial tissue [2, 3]. Traditionally, PCI guidance has relied on angiography; however, evaluating intermediate stenoses of the coronary artery presents a frequent clinical dilemma during coronary angiography. Intermediate stenosis of the coronary artery is defined as an angiographic diameter reduction between 40% and 70% [4]. Angiographic assessment alone has limited accuracy in determining the functional significance of coronary stenoses, which can result in unnecessary stenting of noncritical lesions or deferral of revascularisation in flow‐limiting disease, both of which have negative implications for clinical outcomes and healthcare resource use [5, 6].

To enhance diagnostic accuracy, adjunctive tools, including fractional flow reserve (FFR) and intravascular ultrasound (IVUS), have been integrated into standard clinical practice to overcome the limitations associated with angiography. FFR is a wire‐based physiological index that quantifies the ratio of maximal blood flow in a stenotic artery to theoretical maximal flow in a nondiseased vessel, measured during pharmacologically induced hyperaemia. FFR has been validated in multiple large‐scale trials, including the FAME and FAME‐2 studies, which demonstrated that FFR‐guided PCI reduces the risk of adverse cardiovascular events and avoids unnecessary stenting compared with angiography‐guided strategies, allowing physicians to treat patients safely with medical therapy [7, 8]. Consequently, FFR is recommended in both American and European guidelines as a Class I recommendation in the evaluation and physiological assessment of intermediate lesions [9, 10].

On the other hand, IVUS is a catheter‐based imaging technique that utilises high‐frequency sound waves to generate detailed cross‐sectional and longitudinal images of the coronary artery wall [11]. It allows precise assessment of vessel dimensions, plaque morphology and stent expansion, thereby facilitating accurate lesion characterisation and optimisation of PCI outcomes. IVUS‐guided PCI is associated with improved procedural outcomes, including optimal stent alignment and expansion, as well as reduced rates of stent thrombosis and restenosis, particularly in complex lesions such as those involving the left main or long‐diffuse disease [11, 12]. Although IVUS improves stent optimisation and reduces complications, its adoption remains inconsistent, partly due to higher costs and procedural complexity. European guidelines recommend both FFR and IVUS as Class I indications for the assessment of intermediate coronary lesions [13, 14]. These discrepancies, along with differences in cost‐effectiveness and clinical impact, make it essential to clarify whether anatomical or physiological guidance offers greater benefit in contemporary PCI practice.

FFR and IVUS represent fundamentally different paradigms in PCI guidance. FFR is a physiology‐based tool designed to assess the haemodynamic significance of coronary stenoses, and guide revascularisation decisions based on myocardial ischaemia [7]. In contrast, IVUS is an intravascular imaging modality that provides high‐resolution anatomical characterisation of plaque morphology, vessel dimensions and stent deployment [11]. As such, these modalities address distinct but complementary aspects of coronary intervention: functional lesion assessment versus anatomical refinement. Comparing clinical outcomes between FFR‐guided and IVUS‐guided strategies, therefore, reflects differences in overall revascularisation strategy rather than a direct equivalence of purpose between the two technologies. This conceptual distinction is important when interpreting comparative outcomes.

Beyond the procedural context, the role of coronary revascularisation has been extensively studied in both chronic coronary syndromes (CCS) and acute coronary syndromes (ACS). In CCS, recent meta‐analyses have demonstrated that revascularisation, particularly when guided by physiological assessment, reduces the risk of myocardial infarction and provides superior symptomatic relief compared with medical therapy alone, reinforcing the importance of identifying haemodynamically significant lesions [15–17]. In ACS, timely and complete revascularisation has been shown to improve outcomes, particularly in patients with multivessel disease, where physiologic and imaging guidance can optimise lesion selection and stent deployment [18, 19]. These findings collectively highlight the pivotal role of lesion‐specific assessment and procedural optimisation in improving both short‐ and long‐term outcomes in CAD.

Assessing the comparative efficacy of FFR and IVUS in guiding PCI for intermediate coronary stenoses remains an area of ongoing clinical interest. This systematic review and meta‐analysis is aimed at critically appraising and synthesising current evidence comparing both FFR‐guided PCI and IVUS‐guided PCI in patients with angiographically intermediate coronary lesions.

## 2. Methods

This systematic review and meta‐analysis followed guidance from the Preferred Reporting Items for Systematic Reviews and Meta‐Analyses (PRISMA) statement standard [20]. A study protocol conforming to the PRISMA protocol was registered at the International Prospective Register of Systematic Reviews (PROSPERO ID CRD420251082581) [21] with the PRISMA checklist reported in our Supporting Information.

### 2.1. Eligibility Criteria

This study is aimed at evaluating the comparative outcomes of FFR‐guided PCI and IVUS‐guided PCI in patients with CAD by synthesising available evidence from randomised controlled trials (RCTs) and observational studies.

Studies were included if they directly compared FFR‐guided PCI and IVUS‐guided PCI. Eligible studies had to report at least one primary or secondary outcome relevant to the review. The intervention group consisted of patients undergoing FFR‐guided PCI, whereas the comparator group included those undergoing IVUS‐guided PCI. No restrictions were placed on participant demographics, including age, sex or comorbidities. Only studies published in English were considered for inclusion.

Studies were excluded if they were case reports, single‐arm observational studies without a comparator, narrative reviews or conference abstracts. Additionally, studies that did not report the prespecified outcomes were excluded.

### 2.2. Primary Outcomes

The primary outcome was major adverse cardiovascular events (MACE), defined according to the criteria specified in each individual study. Although the composite endpoint consistently included core components, most commonly all‐cause or cardiac mortality, myocardial infarction and target vessel revascularisation, variations in composite structure, component definitions and adjudication processes were present across studies. In addition to the composite MACE endpoint, individual component outcomes were analysed separately, including all‐cause mortality, cardiac mortality, nonfatal myocardial infarction and target vessel revascularisation.

### 2.3. Secondary Outcomes

The secondary outcomes included the rate of PCI after FFR or IVUS.

### 2.4. Literature Search Strategy

A comprehensive literature search was conducted by two independent reviewers across multiple electronic databases, including MEDLINE (via PubMed), EMBASE, CINAHL, Web of Science and the Cochrane Central Register of Controlled Trials (CENTRAL). The final search was completed on 23 May 2025. The complete search strategies are fully described in the File S1. In addition to database searches, gray literature and ongoing or unpublished trials were identified through searches of the World Health Organization International Clinical Trials Registry Platform (ICTRP), ClinicalTrials.gov and the ISRCTN Register. To ensure thorough coverage, reference lists of all included studies and relevant systematic reviews were manually screened for additional eligible articles.

### 2.5. Study Selection

Two independent reviewers, Z.A. and K.F. (blinded to each other′s assessments), screened all retrieved studies for eligibility based on titles and abstracts. Full‐text articles were obtained for studies that appeared to meet the inclusion criteria. If discrepancies in the selection process were identified, they were resolved through discussion or consultation with a third reviewer, E.J.

### 2.6. Data Extraction and Management

A standardised data extraction spreadsheet was created in Microsoft Excel, based on the Cochrane data collection form for intervention reviews. The spreadsheet underwent pilot testing to ensure consistency and reliability. Two independent reviewers extracted data from the included studies, and any disagreements were resolved through discussion. The extracted data included study characteristics (author, year, country, study design and sample size), patient characteristics (mean age, sex distribution and relevant clinical background), intervention details (FFR threshold values, IVUS criteria for PCI and specific procedural guidance strategies) and all prespecified primary and secondary outcomes.

### 2.7. Data Synthesis

A meta‐analysis was conducted for outcomes reported by at least four studies. Mean differences (MDs) were used to assess continuous variables, whereas risk ratios (RRs) were calculated for dichotomous outcomes. A random‐effect model was applied for statistical analysis. In addition to relative effect estimates, absolute event rates and absolute risk differences (ARDs) with corresponding 95% confidence intervals (CIs) were calculated from pooled event totals to enhance clinical interpretability. Where appropriate, numbers needed to treat or harm (NNT/NNH) were derived from statistically significant absolute risk differences. Review Manager 5.3 (RevMan) software was used for the primary meta‐analyses, and results were presented as forest plots with 95% CIs. The degree of heterogeneity among studies was evaluated using Cochrane′s *Q* test (*χ* ^2^) and the *I* ^2^ statistic. The *I* ^2^ statistic was interpreted as follows: 0%–25% indicating low heterogeneity, 25%–75% moderate heterogeneity and 75%–100% high heterogeneity. Subgroup analyses were performed according to study design (RCTs vs. observational studies). Subgroup analysis according to FFR decision threshold (≤ 0.80 vs. < 0.75) was prespecified to explore the potential impact of physiological cut‐off variability on pooled estimates. However, as only one study applied a < 0.75 threshold, statistically robust subgroup comparison was not feasible.

### 2.8. Risk of Bias and Quality Assessment

The methodological quality of RCTs was assessed using the Cochrane risk‐of‐bias tool, and observational studies were evaluated using the Newcastle–Ottawa scale. Studies were further classified according to AHRQ criteria. Two reviewers assessed quality independently, with disagreements resolved by consensus. Certainty of evidence for each primary outcome (MACE, all‐cause mortality, cardiac death, nonfatal myocardial infarction and target vessel revascularisation) was assessed using the GRADE (Grading of Recommendations, Assessment, Development and Evaluation) approach, evaluating risk of bias, inconsistency, indirectness, imprecision and publication bias. Overall certainty was classified as *high*, *moderate*, *low* or *very low*.

## 3. Results

### 3.1. Literature Search Results

**Figure 1:**
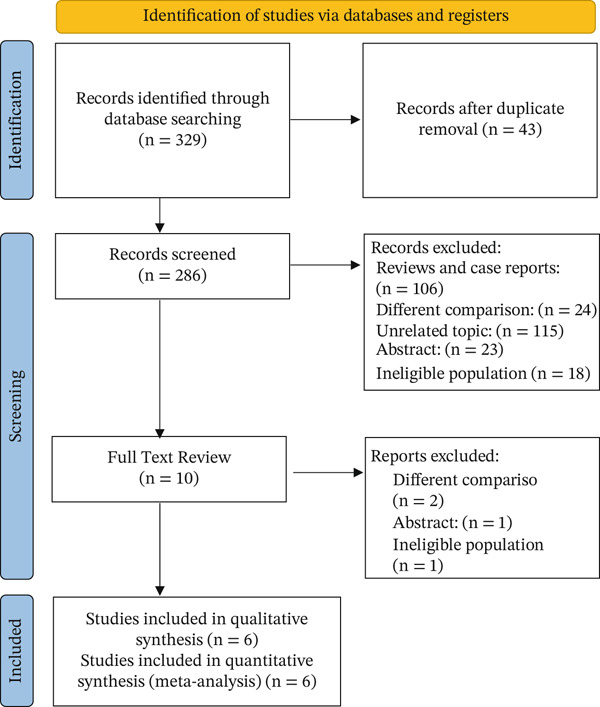
PRISMA flow chart.

Our search strategy retrieved six studies. After screening the retrieved articles, the authors identified six studies that met the eligibility criteria. (Figure 1).

### 3.2. Description of the Studies

Six studies were appraised, including two RCTs and four observational cohorts, with a total of 5040 patients [22–27]. Of these, 2517 patients underwent FFR‐guided PCI and 2523 underwent IVUS‐guided PCI. Table 1 summarises the characteristics of the included studies. Table 2 summarises the baseline characteristics of the patient cohort included in the studies. The mean age ranged from 62 to 67 years, and male patients accounted for 60%–75% of the cohorts. Hypertension was the most common comorbidity (60%–70%), followed by diabetes mellitus (15%–30%) and dyslipidaemia (> 65%). Smoking prevalence ranged between 15% and 30%, whereas chronic kidney disease was reported in up to 25% of patients in selected studies. Approximately one‐third of patients presented with ACSs, whereas the remainder had stable coronary disease. Left ventricular systolic function was generally preserved, with a mean ejection fraction of 55%–60%. Prior myocardial infarction and previous revascularisation (PCI or CABG) were variably reported across studies. Overall, baseline profiles were broadly comparable between the FFR‐ and IVUS‐guided groups. Procedural optimisation criteria, including stent expansion indices, post‐PCI physiological targets and reporting of intraprocedural complications, varied across studies and are summarised in Tables 3 and 4.

**Table 1:** Characteristics of included studies. FFR, fractional flow reserve; IVUS, intravascular ultrasound; NSTE‐ACS, non‐ST segment–elevation acute coronary syndrome; PCI, percutaneous coronary intervention; MSA, minimal stent area; TV‐MI, target vessel–related spontaneous myocardial infarction; TVR, target vessel revascularization; MLA, minimal lumen area; MI, myocardial infarction; RCT, randomised controlled trial; MACE, major adverse cardiac event; non‐LM, nonleft main coronary artery; TLR, target lesion revascularisation.

Author	Sample size	Study design	Inclusion criteria	Indications for PCI	Follow‐up duration	Follow‐up completion	Outcome of interest
Budrys et al. (2023)	FFR: 74 IVUS: 80	Retrospective observational cohort	Over 18, diagnosed with chronic coronary syndrome or NSTE‐ACS, haemodynamically significant lesions necessitating a stent length of 30 mm or more and suitable for PCI.	FFR: (FFR ≤ 0.80) or anatomical IVUS: achieving adequate stent expansion (with an MSA greater than 90% of the distal reference lumen area and/or MSA of at least 5.5 mm^2^), maintaining a plaque burden below 50% within 5 mm proximally and distally to the stent.	9–12 months	126/154	Target vessel failure rate, (target‐vessel‐related death [TV‐death], TV‐MI, any TVR) during 1‐year follow‐up; functional target vessel restenosis rate at 9–12 months follow‐up.
Wu et al. (2025)	FFR: 202 IVUS: 196	Prospective, nonrandomised, sequential cohort study	Proximal and midsegment coronary lesions with diameters exceeding 2.5 mm, and the stenosis degree ranged from 40% to 70%.	IVUS: MLA < 4 mm^2^, FFR: FFR < 0.80.	1, 3, 6 and 12 months	24 lost to follow‐up	Medicine use and adverse events, MI, death, TVR.
Hu et al. (2025)	FFR: 923 IVUS: 916	RCT	Enrolled patients aged 18 years or older with suspected ischaemic heart disease and with at least 50% stenosis in epicardial coronary arteries measuring at least 2·5 mm by visual estimation on coronary angiography.	FFR: FFR 0.80 or less IVUS: MLA measuring 3 mm^2^ or less or an MLA measuring 3–4 mm^2^, with a plaque burden of more than 70%.	12 months	FFR: 909/923, IVUS: 905/916	The primary outcome was a patient‐oriented composite outcome of death, MI, or any revascularisation by 12 months after randomisation.
Nam et al. (2010)	FFR: 80 IVUS: 87	Retrospective observational cohort	De novo intermediate coronary stenosis (defined as 40%–70% diameter stenosis by visual assessment).	FFR ≤ 0.80 or IVUS MLA ≤ 4 mm2.	1 year	FFR: 98.8%, IVUS: 97.9%	MACE: composite of all‐cause death, nonfatal MI, ischemia‐driven TVR.
Hernandez et al. (2013)	FFR: 400 IVUS: 400	Retrospective observational cohort	Intermediate angiographic stenosis (40%–70%); de novo non–LM lesions; assessment by FFR or IVUS.	1) FFR < 0.75 2) MLA < 4 and OR < 3.5 mm^2^ + plaque burden > 50*%*.	1 and 2 years	At 1 year: 97.7% both groups. At 2 years: 93.1% (FFR) and 95.6% (IVUS)	MACE, including cardiac death, target lesion MI and TLR at 12 months.
Koo et al. (2022)	FFR: 838 IVUS: 844	RCT	NR.	NR.	24 months	99.20%	NR.

**Table 2:** Patient characteristics at baseline. FFR, fractional flow reserve; IVUS, intravascular ultrasound; BMI, body mass index; CKD, chronic kidney disease; ACS, acute coronary syndromes; STEMI, ST‐segment elevation myocardial infarction; NSTEMI, nonST segment–elevation myocardial infarction; LVEF, left ventricular ejection fraction; MI, myocardial infarction; PCI, percutaneous coronary intervention; CABG, coronary artery bypass graft; NR, not reported.

	Budrys et al. (2023)	Wu et al. (2025)	Hu et al. (2025)	Nam et al. (2010)	Hernandez et al. (2013)	Koo et al. (2022)
Mean age ± SD (range)	FFR: 66.3 ± 9.6 IVUS: 66.2 ± 9.0	FFR: 62 ± 17 IVUS: 67 ± 18	FFR: 66·0 (58–72) IVUS: 66 (58–72)	FFR: 63 +/−9. IVUS: 62+/−9	FFR: 65.9 ± 9.5 IVUS: 65.2 ± 10	FFR: 65.4 ± 9.4 IVUS: 64.8 ± 9.9
Sex (male: female)	FFR: 54:20 IVUS: 57:23	FFR: 124:78 IVUS: 110:86	FFR: 624: 299 IVUS: 624:292	FFR: 55: 28. IVUS: 55:39	FFR: 297: 103 IVUS: 298:102	FFR: 584:254 IVUS: 603:241
BMI	NR	NR	FFR: 24.2(22.2–26.4) IVUS: 24.1 (22.2–26.5)	NR	NR	NR
Hypertension	FFR = 67/74 IVUS = 74/80	FFR: 104 IVUS: 108	FFR = 615/923 IVUS = 628/916	FFR: 35/83 IVUS: 48/94	FFR: 291/400 IVUS: 280/400	FFR: 577/838 IVUS: 570/844
Diabetes	FFR = 16/74 IVUS = 15/80	FFR: 60 IVUS: 54	FFR = 279/923 IVUS = 290/916	FFR: 18/83 IVUS: 24/94 ^∗^83 and 94 are total number of lesions, not patients	FFR: 159/400 IVUS: 150/400	FFR: 272/838 IVUS: 282/844
Hypercholesterolaemia	NR	FFR: 122 IVUS: 118	NR	FFR: 13/83 IVUS: 14/94	FFR: 214/400 IVUS: 234/400	NR
Dyslipidaemia	FFR: 67/74 IVUS: 72/80	NR	FFR: 616/923 IVUS: 614/916	NR	NR	FFR: 667/838 IVUS: 655/844
Current smokers	FFR: 15/74 IVUS = 20/80	FFR: 108 IVUS: 118	FFR: 242/923 IVUS: 235/916	FFR: 27/83 IVUS: 34/94	FFR: 102/400 IVUS: 85/400	FFR: 166/838 IVUS: 155/844
CKD	FFR: 12/74 IVUS = 16/80	NR	FFR: 210/923 IVUS: 237/916	NR	NR	FFR: 143/838 IVUS: 147/844
Stable angina	NR	FFR: 158 IVUS: 150	NR	FFR: 38/83 IVUS: 34/94	FFR: 126/400 IVUS: 120/400	FFR: 519/838 IVUS: 544/844
ACS	NR	FFR: 44 IVUS: 46	FFR: 545 IVUS: 542	FFR: 45/83 IVUS: 60/94	NR	FFR: 252/838 IVUS: 244/844
STEMI	NR	NR	FFR: 9 IVUS: 9	NR	FFR: 36/400 IVUS: 46/400	FFR: 4/838 IVUS: 4/844
NSTEMI	NR	NR	FFR: 48 IVUS: 33	NR	FFR: 216/400 IVUS: 209/400	FFR: 12/838 IVUS: 15/844
Chronic coronary syndrome	NR	NR	FFR: 371 IVUS: 365	NR	NR	NR
Silent ischaemia	NR	NR	NR	NR	FFR: 22/400 IVUS: 24/400	NR
LVEF (%)	FFR: 50.0 ± 5 IVUS: 55.0 ± 5	FFR: 57 ± 7 IVUS: 55 ± 6	FFR: 64.9% (60.0–69.3) IVUS = 64.0*%* (60.0–69.1)	FFR: 61 +/−10 IVUS: 59 +/−10	$\underset{\_}{\text{LVEF}<45\%}$ $\underset{\_}{\left(n\right)=}\;\text{FFR}$: 63/400 IVUS: 78/400	FFR: 63.3 ± 8.5 IVUS: 63.9 ± 8.3
Previous MI	FFR: 29/74 IVUS: 46/80	NR	FFR: 129/923 IVUS: 126/916	NR	FFR: 78/400 IVUS: 80/400	FFR: 56/838 IVUS: 39/844
Previous PCI	NR	FFR: 78/202 IVUS: 58/196	FFR: 300/923 IVUS: 259/916	NR	FFR: 93/400 IVUS: 76/400	FFR: 165/838 IVUS: 163/844
Previous CABG	FFR: 2/74 IVUS: 1/80	NR	NR	NR	FFR: 4/400 IVUS: 9/400	NR

**Table 3:** Procedural characteristics pre‐ and post‐PCI. FFR, fractional flow reserve; IVUS, intravascular ultrasound; PCI, percutaneous coronary intervention; LAD, left anterior descending artery; LCx, left circumflex artery; RCA, right coronary artery; MLD, minimal lumen diameter; MLA, minimal lumen area; MSA, minimal stent area; SYNTAX, synergy between percutaneous coronary intervention with taxus and cardiac surgery score; NR, not reported.

	Budrys et al. (2023)	Wu et al. (2025)	Hu et al. (2025)	Nam et al. (2010)	Hernandez et al. (2013)	Koo et al. (2022)
Extent of disease (single vs. multivessel)	Single vessel = FFR: 11/74 IVUS: 12/80 Two − vessel = FFR: 39/74 IVUS: 35/80 Three − vessel = FFR: 24/74 IVUS: 33/80	Multivessel = FFR: 112/202 IVUS: 110/196	NR	Multivessel = FFR: 55/83. IVUS:46/94	Multivessel = FFR:90/400 IVUS: 81/400	Multivessel = FFR: 445/838 IVUS:430/844
Target vessel (LAD vs. non‐LAD)	LAD = FFR: 61/74 IVUS:66/80 LCx = FFR: 8/74 IVUS: 7/80 RCA = FFR: 5/74 IVUS:7/80	LAD = FFR: 104/202 IVUS: 88/196	LAD = FFR: 565/990 IVUS: 606/984 LCx = FFR: 146/990 IVUS: 130/984 RCA = FFR: 279/990 IVUS:248/984	LAD = FFR: 40/83. IVUS:55/94	LAD = FFR:265/463 IVUS:280/488 LCx = FFR:88/463 IVUS:79/488 RCA = FFR: 110/463 IVUS: 129/488	LAD = 61.9*%*
PCI involving left main artery	FFR: 2/74 IVUS: 13/80	NR	NR	NR	NR	NR
Lesion location (proximal vs. mid)	NR	Proximal = FFR: 106/202 IVUS: 94/196	NR	Proximal = FFR: 40/83 IVUS: 43/94	NR	NR
Lesion type (simple vs. complex)	NR	NR	NR	Simple = FFR: 25/83. IVUS: 30/94	NR	NR
Reference vessel diameter (mm)	NR	NR	FFR: 2·93 (2·61–3·30) IVUS: 2·96 (2·65–3·35)	FFR: 3.23 ± 0.43 IVUS: 3.39 ± 0.49	FRR: 3 ± 0.5 IVUS: 3.05 ± 0.4	FFR: 3.11 ± 0.43 IVUS: 3.19 ± 0.43
Lesion length (mm)	NR	FFR: 25 ± 12 IVUS: 22 ± 13	FFR: 19·0 (12·5–29·8) IVUS: 20·3 (13·2–30·5)	FFR: 24+/−12 IVUS: 24+/−13	FFR: 14 ± 9 IVUS: 13.5 ± 10	FFR: 16.5 ± 24.1 IVUS: 25.2 ± 28.1
Average stent diameter	FFR: 3.25 IVUS: 3.25	NR	NR	NR	NR	NR
% Diameter stenosis at baseline	NR	FFR: 55 ± 10 IVUS: 53 ± 8	FFR: 62.3% (53·8–70·9) IVUS: 62·2% (54·7–70·0)	FFR: 51 +/−8 IVUS: 52 +/−8	FFR: 49.5 ± 9 IVUS: 50.5 ± 10	FFR: 56.7 ± 10.1 IVUS: 56.9 ± 10.1
%Diameter stenosis postintervention	NR	NR	NR	NR	FFR: 11 ± 4 IVUS: 11 ± 3	NR
MLD at baseline (mm)	IVUS: 1.8 ± 0.2	NR	FFR: 1·10 (0·82–1·38) IVUS: 1·12 (0·83–1·39)	FFR: 1.59 ± 0.32 IVUS: 1.61 ± 0.45	NR	NR
Postintervention MLD (mm)	NR	NR	NR	FFR: 2.89 ± 0.42 IVUS: 3.03 ± 0.47	NR	NR
FFR (baseline and post‐PCI)	Baseline = FFR: 0.63 IVUS: 0.66 $\underset{\_}{\text{Post}-\text{PCI}}=\text{FFR}$: 0.88 IVUS: 0.88	Baseline = defer group: 0.88 ± 0.07 PCI group: 0.71 ± 0.06Post − PCI = 0.93 ± 0.05	Baseline = onsite: 0·73 (0·56–0·84) Core laboratory: 0·74 (0·57–0·86) Post − PCI = onsite: 0·96 (0·93–0·98) Core laboratory: 0·95 (0·93–0·97)	Baseline = defer: 0.87 ± 0.06. PCI: 0.72 ± 0.07Post − PCI = 0.91 ± 0.05	Baseline = 0.82 ± 0.09	Baseline = 0.83 ± 0.09Post − PCI = 0.88 ± 0.06
Preinterventional MLA (for IVUS) mm^2^	IVUS: 2.5 ± 0.6	IVUS = defer: 3.60 ± 0.67 PCI: 3.12 ± 0.49	Onsite = IVUS: 2·68 (2·19–3·35) Core laboratory = IVUS: 2·55 (2·07–3·36)	Defer: 5.1 ± 1.5 PCI: 2.9 ± 0.9	4.22 ± 1	3.4 ± 1.3
Postinterventional MSA (for IVUS) mm^2^	IVUS: 5.9 ± 1.9	IVUS: 7.21 ± 0.57	Onsite = IVUS: 6·76 (5·49–8·57) $\text{Core}\;\text{laboratory}\;\underset{\_}{=}\;\text{IVUS}$: 5·80 (4·72–7·25)	7.3 ± 2.8	NR	7.0 ± 2.2
Plaque burden (%)	IVUS: plaque < 50*%* = 56/80	IVUS = defer: 63.2 ± 8.5 PCI: 70.3 ± 7.4	Onsite = IVUS: 76·0% (70·0–81·0) Core laboratory = IVUS: 76·1% (69·7–81·2)	NR	NR	70.1 ± 10.2
SYNTAX score	NR	NR	Baseline = FFR: 10 (5–15) IVUS: 9 (5–15) After PCI = FFR: 5 (0–9) IVUS: 5 (0–8)	NR	NR	Baseline = FFR: 8.4 ± 5.8 IVUS: 8.9 ± 6.2Post − PCI = FFR: 5.4 ± 4.6 IVUS: 4.6 ± 4.7
Total stent length (mm)	FFR: 49.0 IVUS: 62.5	FFR: 22 ± 8.6 IVUS: 24 ± 9.3	NR	NR	NR	NR
Average stent diameter	FFR: 3.25 IVUS: 3.25	FFR: 2.90 ± 0.30 IVUS: 2.92 ± 0.33	NR	NR	NR	NR
Minimal stent diameter (mm)	IVUS: 2.5 ± 0.4	NR	FFR: 2·61 (2·35–2·93) IVUS: 2·66 (2·42–2·96)	NR	NR	NR

**Table 4:** This table summarises procedural optimisation criteria and intraprocedural endpoints reported in studies comparing fractional flow reserve (FFR)– and intravascular ultrasound (IVUS)–guided percutaneous coronary intervention (PCI). Reporting standards varied across studies. Abbreviations: FFR, fractional flow reserve; IVUS, intravascular ultrasound; PCI, percutaneous coronary intervention; MSA, minimal stent area; MLA, minimal lumen area; LAD, left anterior descending artery; LCx, left circumflex artery; RCA, right coronary artery; MLD, minimal lumen diameter; SYNTAX, Synergy Between Percutaneous Coronary Intervention With Taxus and Cardiac Surgery score; NR, not reported.

Study	FFR Threshold for PCI	Post‐PCI FFR target/reported	IVUS criteria for PCI	IVUS optimisation/Stent expansion criteria	Stent expansion index reported	Procedural complications reported
Nam et al. (2010)	≤ 0.80	Not specified	MLA < 4.0 mm^2^	No predefined expansion index	No	Reported within composite outcomes
Hernandez et al. (2013)	< 0.75	Not specified	MLA < 4.0 mm^2^ (> 3 mm vessel) or <3.5 mm^2^ (2.5–3 mm vessel) + plaque burden > 50*%*	No formal optimisation index reported	No	Not systematically reported
Koo et al. 2022	≤ 0.80	≥ 0.88 or *Δ* *F* *F* *R* <0.05	MLA ≤ 3 mm^2^ or 3–4 mm^2^ + plaque burden > 70*%*	Minimal stent area ≥ 5.5 mm^2^ or ≥ distal reference lumen	Yes (core‐lab defined minimal stent area criteria)	Systematically reported
Hu et al. (2025)	≤ 0.80 (angiography‐derived FFR)	Prespecified physiological optimisation targets	Prespecified IVUS criteria	Protocol‐defined IVUS optimisation	Yes (protocol‐defined)	Systematically reported
Budrys et al. (2023)	≤ 0.80	Post‐PCI FFR measured; ≤ 0.80 considered suboptimal	Long‐lesion protocol	No standardised expansion index reported	No	Limited reporting
Wu et al. (2025)	< 0.80	Not specified	MLA < 4.0 mm^2^	No formal optimisation index reported	No	Limited reporting

### 3.3. Primary Outcomes

#### 3.3.1. MACE Events

**Figure 2:**
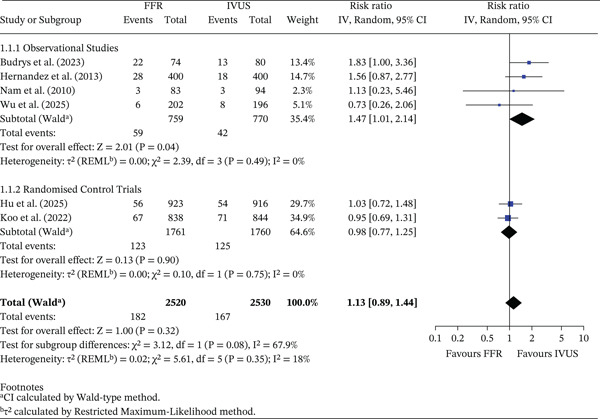
Forest plot of FFR versus IVUS for guiding PCI—number of MACE events outcome.

There was no overall difference in MACE between FFR‐ and IVUS‐guided PCI (RR = 1.13, 95% CI 0.89–1.44, *p* = 0.32; *I* ^2^ = 18*%*). The pooled absolute MACE rate was 7.22% (182/2520) in the FFR group and 6.60% (167/2530) in the IVUS group, corresponding to an absolute risk difference (ARD) of −0.62% (95% CI −2.02% to 0.78%). In subgroup analyses, observational studies showed a higher relative risk of MACE with FFR (RR = 1.47, 95% CI 1.01–2.14, *p* = 0.04; *I* ^2^ = 0*%*), with absolute event rates of 7.77% (FFR) and 5.45% (IVUS), yielding an ARD of −2.32% (95% CI −4.81% to 0.17%). In contrast, RCTs demonstrated no difference between strategies (RR = 0.98, 95% CI 0.77–1.25, *p* = 0.90; *I* ^2^ = 0*%*), with absolute rates of 6.98% (FFR) and 7.10% (IVUS) (ARD 0.12%, 95% CI −1.57% to 1.81%). The test for subgroup differences was not significant (*p* = 0.08), suggesting no clear influence of study design on observed outcomes. This is shown in Figure 2.

#### 3.3.2. All‐Cause Death

**Figure 3:**
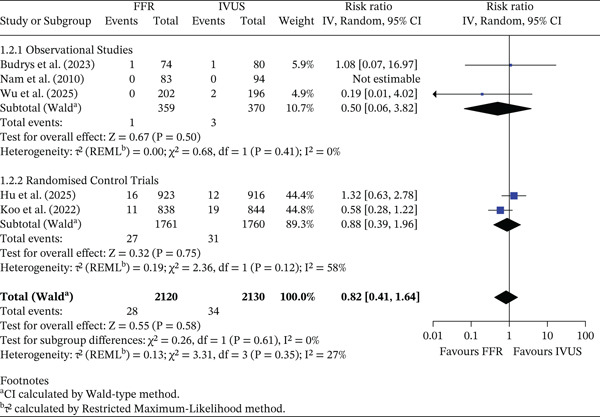
Forest plot of FFR versus IVUS for guiding PCI—number of all‐cause death events.

No significant difference was observed in all‐cause mortality between FFR‐ and IVUS‐guided PCI (RR = 0.82, 95% CI 0.41–1.64, *p* = 0.58; *I* ^2^ = 27*%*). The pooled absolute mortality rate was 1.32% (28/2120) in the FFR group and 1.60% (34/2130) in the IVUS group, corresponding to an ARD of 0.28% (95% CI −0.48% to 1.04%). In observational studies, mortality rates were 0.28% (FFR) and 0.81% (IVUS), yielding an ARD of 0.53% (95% CI −0.53% to 1.59%). Among RCTs, absolute rates were 1.53% (FFR) and 1.76% (IVUS) (ARD 0.23%, 95% CI −0.64% to 1.10%). The test for subgroup differences was not statistically significant (*p* = 0.61), indicating no evidence that study design modified the effect. This is shown in Figure 3.

#### 3.3.3. Cardiac‐Related Death

**Figure 4:**
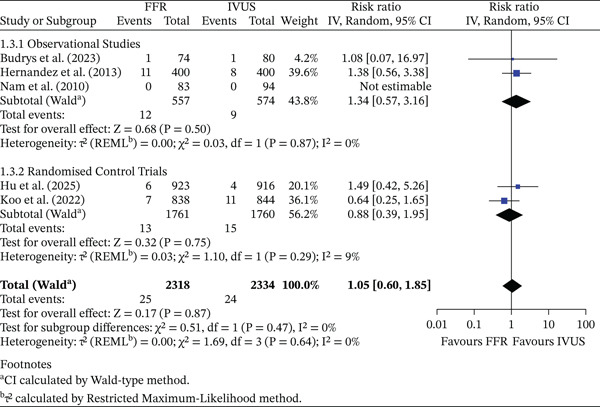
Forest plot of FFR versus IVUS for guiding PCI—number of cardiac‐related death events.

There was no significant difference in cardiac mortality between FFR‐ and IVUS‐guided PCI (RR = 1.05, 95% CI 0.60–1.85, *p* = 0.87; *I* ^2^ = 0*%*). The pooled absolute event rate was 1.08% (25/2318) in the FFR group and 1.03% (24/2334) in the IVUS group, corresponding to an ARD of −0.05% (95% CI −0.64% to 0.54%). In observational studies, cardiac mortality rates were 2.15% (FFR) and 1.57% (IVUS), yielding an ARD of −0.58% (95% CI −2.23% to 1.07%). RCTs showed absolute rates of 0.74% (FFR) and 0.85% (IVUS) (ARD 0.11%, 95% CI −0.50% to 0.72%). The test for subgroup differences was not statistically significant (*p* = 0.47). This is shown in Figure 4.

#### 3.3.4. Nonfatal Myocardial Infarction

**Figure 5:**
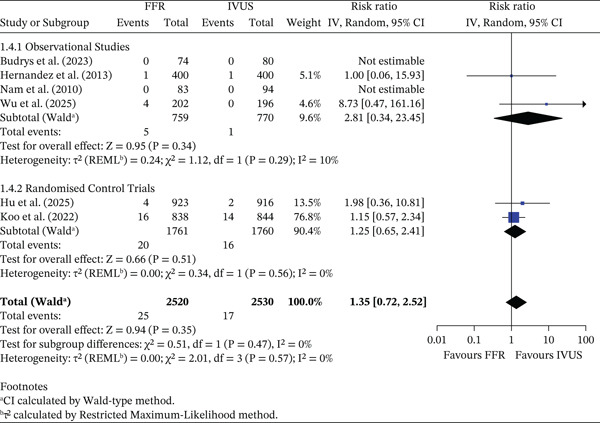
Forest plot of FFR versus IVUS for guiding PCI–number of nonfatal myocardial infarction events.

There was no significant difference in the risk of nonfatal myocardial infarction between FFR‐ and IVUS‐guided PCI (RR = 1.35, 95% CI 0.72–2.52, *p* = 0.35; *I* ^2^ = 0*%*). The pooled absolute rate was 0.99% (25/2520) in the FFR group and 0.67% (17/2530) in the IVUS group, corresponding to an ARD of −0.32% (95% CI −0.82% to 0.18%). In observational studies, absolute rates were 0.66% (FFR) and 0.13% (IVUS) (ARD −0.53%, 95% CI −1.16% to 0.10%). In RCTs, rates were 1.14% (FFR) and 0.91% (IVUS) (ARD −0.23%, 95% CI −0.89% to 0.44%). The test for subgroup differences was not significant (*p* = 0.47). This is shown in Figure 5.

#### 3.3.5. Target Vessel Revascularisation

**Figure 6:**
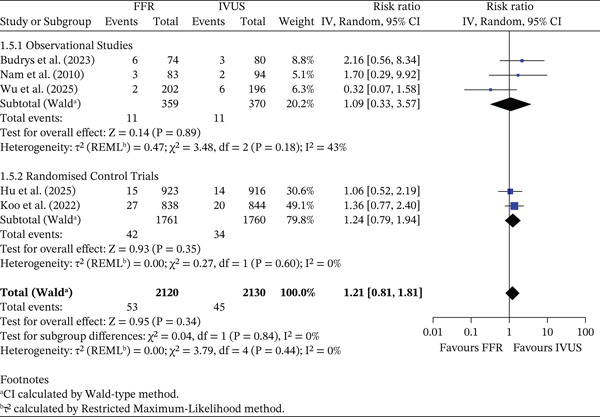
Forest plot of FFR versus IVUS for guiding PCI—number of TVR events.

There was no significant difference in the risk of TVR between FFR‐ and IVUS‐guided groups (RR = 1.21, 95% CI 0.81–1.81, *p* = 0.34; *I* ^2^ = 0*%*). The pooled absolute TVR rate was 2.50% (53/2120) in the FFR group and 2.11% (45/2130) in the IVUS group, corresponding to an ARD of −0.39% (95% CI −1.29% to 0.52%). In observational studies, rates were 3.06% (FFR) and 2.97% (IVUS) (ARD −0.09%, 95% CI −2.58% to 2.39%). In RCTs, rates were 2.39% (FFR) and 1.93% (IVUS) (ARD −0.45%, 95% CI −1.41% to 0.51%). The test for subgroup differences was not significant (*p* = 0.84). This is shown in Figure 6.

### 3.4. Secondary Outcomes

#### 3.4.1. PCI Procedures

**Figure 7:**
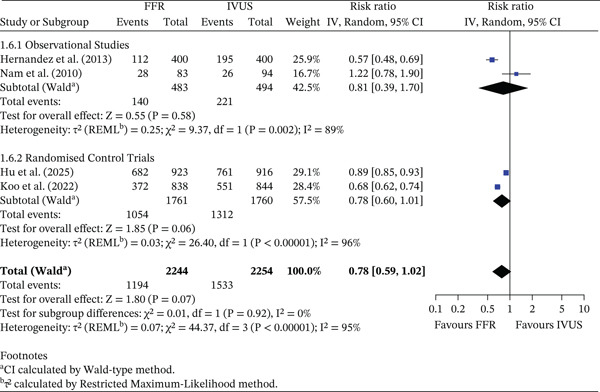
Forest plot of FFR versus IVUS for guiding PCI—number of postimaging PCI events.

There was no statistically significant difference in the relative risk of PCI procedures completed between FFR‐ and IVUS‐guided groups (RR = 0.78, 95% CI 0.59–1.02, *p* = 0.07). However, the pooled absolute PCI rate was 53.2% (1194/2244) in the FFR group and 68.0% (1533/2254) in the IVUS group, corresponding to an ARD of 14.84% (95% CI 11.92%–17.76%). This translates to approximately one additional PCI performed for every seven patients managed with IVUS guidance compared with FFR. Observational studies demonstrated absolute rates of 28.99% (FFR) and 44.74% (IVUS) (ARD 15.75%, 95% CI 9.71%–21.79%), whereas RCTs showed rates of 59.85% (FFR) and 74.55% (IVUS) (ARD 14.70%, 95% CI 11.56%–17.84%). Substantial heterogeneity was observed (*I* ^2^ = 95*%*). The test for subgroup differences was not significant (*p* = 0.92). This is shown in Figure 7.

#### 3.4.2. Methodological Quality and Risk‐of‐Bias Assessment

**Figure 8:**
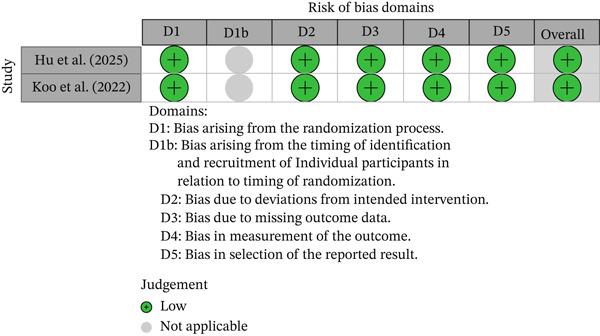
Risk of bias assessment using the Cochrane risk‐of‐bias tool for RCTs.

The quality of the randomised studies was assessed using the Cochrane Collaboration tool. This is summarised in Figure 8. The observational studies were evaluated using the Newcastle–Ottawa scale, summarised in Table 5. According to the Agency for Healthcare Research and Quality standards, all the studies were rated to be of good quality. The overall certainty of evidence ranged from low to moderate across primary outcomes (Table 6), with downgrading mainly due to observational study design, heterogeneity between studies, and possible publication bias.

**Table 5:** Newcastle–Ottawa scale to assess the quality of nonrandomised studies.

Author (year)	Selection (out of four stars)	Comparability (out of two stars)	Outcome/exposure (out of three stars)	Quality assessment based on the Agency for Healthcare Research and Quality (AHRQ)
Wu et al. (2025)	∗∗∗	∗	∗∗∗	Fair
Budrys et al. (2023)	∗∗∗	∗	∗∗∗	Fair
Hernandez et al. (2013)	∗∗∗∗	∗∗	∗∗∗	Good
Nam et al. (2010)	∗∗∗	∗	∗∗∗	Fair

**Table 6:** Certainty of evidence for primary outcomes comparing FFR‐guided and IVUS‐guided PCI, assessed using the GRADE (Grading of Recommendations, Assessment, Development and Evaluation) framework across risk of bias, inconsistency and indirectness. “Mixed” indicates inclusion of both RCTs and observational studies. MACE: major adverse cardiovascular events; TVR: target vessel revascularisation.

Outcome	Number of studies	Study design	Risk of bias	Inconsistency	Indirectness
MACE		Mixed	Moderate	Moderate	Low
All‐cause mortality	5	Mixed	Moderate	Low	Low
Cardiac death	5	Mixed	Moderate	Low	Low
Nonfatal myocardial infarction	6	Mixed	Moderate	High	Low
TVR	5	Mixed	Moderate	High	Low

## 4. Discussion

This systematic review compared both the procedural and clinical outcomes of FFR‐guided PCI and IVUS‐guided PCI among patients exhibiting angiographically intermediate coronary artery stenoses. The findings of this study demonstrate an absence of statistically significant differences in MACE and its components: all‐cause mortality, cardiac‐related death, nonfatal MI and TVR, when evaluating both strategies, although the evidence remains limited and heterogeneous. Subgroup analyses by study design did not show clear evidence of effect modification, although observational data suggested a higher risk of MACE in FFR‐guided patients, a finding not replicated in RCTs. Based on the current available literature, this is the first systematic review and meta‐analysis to directly compare the outcomes of both FFR and IVUS, encompassing the FLAVOUR and FLAVOUR II trials.

In contrast, IVUS is primarily an imaging modality used after the decision to perform PCI has been made, with its main role being the optimisation of stent implantation and enhancement of long‐term clinical outcomes. Although IVUS can assist in lesion assessment, its major contribution lies in ensuring optimal stent expansion, apposition and detection of edge dissections or underdeployment, factors that have been strongly associated with improved long‐term prognosis [28, 29].

The findings indicated comparable hard clinical outcomes of adverse cardiovascular events after both procedures, which is consistent with existing literature and guidelines that both FFR and IVUS offer similar outcomes and benefits. FFR provides a physiological assessment that enables lesion‐specific revascularisation, frequently allowing PCI in nonischaemic stenoses and, hence, reducing overtreatment. Overtreatment with PCI may expose patients to unnecessary procedural risks, increased healthcare costs and higher rates of long‐term complications such as in‐stent restenosis or stent thrombosis, without improving clinical outcomes [14]. In contrast, IVUS is primarily an imaging modality used after the decision to perform PCI has been made, with its main role being the optimisation of stent implantation and enhancement of long‐term clinical outcomes. These are particularly beneficial in complex lesions, including those involving the left main stem, bifurcations and long‐diffuse disease [30, 31]. Although IVUS can assist in lesion assessment, its major contribution lies in ensuring optimal stent expansion, apposition and detection of edge dissections or underdeployment, factors that have been strongly associated with improved long‐term prognosis [28, 29].

Our pooled analysis from six studies indicated no statistically significant difference in nonfatal myocardial infarction or TVR between the FFR‐ and IVUS‐guided groups. Although these differences were not statistically significant, they may reflect a higher risk of undertreatment where revascularisation is deferred in lesions deemed functionally nonsignificant by FFR despite vulnerability and anatomical complexity. Conversely, IVUS‐guided PCI may reduce the incidence of such events by enabling more comprehensive plaque assessment and optimal stent expansion, translating into improved stent durability and reduced repeat revascularisation, as supported by recent studies [11]. The American College of Cardiology (ACC) and American Heart Association (AHA) guidelines for coronary artery revascularisation assign a Class I recommendation to FFR for the evaluation of angiographically intermediate coronary stenoses [9]. In contrast, IVUS is recommended with a Class IIa indication for anatomical optimisation of stent deployment in complex PCI [9]. However, the 2024 European Society of Cardiology (ESC) guidelines endorse both FFR and IVUS as Class I recommendations for physiological and anatomical guidance, underscoring their complementary rather than competing roles in PCI. [10].

The frequency of PCI procedures conducted was similar in the FFR‐guided group compared with the IVUS‐guided group, indicating that both strategies led to comparable revascularisation rates despite their differing approaches to lesion assessment. However, the high heterogeneity in this result (*I* ^2^ = 95*%*) suggests the presence of differences in study designs, methodologies, operator training and experience and FFR protocols, which may influence the generalisability and effect of these results on a larger scale. Therefore, we recommend large RCTs to be conducted in the future to strengthen the evidence base.

A crucial consideration in interpreting the findings is the impact of operator experience and the associated learning curve for both FFR and IVUS. The effectiveness of both physiology‐ and imaging‐guided strategies may be influenced by operator expertise and institutional familiarity with each modality. Variability in technical execution and interpretation may partly explain differences in procedural decisions across studies.

Although cost considerations may influence the choice of intracoronary guidance modality in certain healthcare systems, formal cost‐effectiveness analysis was beyond the scope of this study. Decisions regarding the use of IVUS or FFR are therefore likely to depend on institutional resources, procedural complexity and operator expertise rather than clear differences in hard clinical outcomes. Future dedicated health economic analyses are required to clarify comparative cost‐effectiveness.

In addition, the certainty of evidence for the primary outcomes, assessed using the GRADE framework, was moderate for MACE and all‐cause mortality and low for cardiac death, nonfatal MI and TVR. This grading reflects the inclusion of observational data, moderate heterogeneity between studies and imprecision arising from small event numbers. Despite rigorous methodology, residual confounding cannot be excluded, as variations in patient selection, operator expertise and procedural techniques may have influenced both procedural and clinical outcomes.

### 4.1. Limitations

Despite comprising a meta‐analysis with high‐quality and large observational studies, considerable heterogeneity in study design, patient populations and procedural protocols may affect the validity of the findings. Also, reporting bias may have been introduced, as studies included varying definitions of MACE. Importantly, both FFR‐ and IVUS‐guided strategies are inherently operator‐dependent techniques with notable learning curves. Accurate physiological measurement, plaque interpretation, stent landing zone selection and optimisation of stent expansion require technical expertise and institutional familiarity. Variability in procedural volume, training and adherence to optimisation protocols was not consistently reported across included studies and could not be adjusted for in this aggregate meta‐analysis. Differences in operator proficiency may therefore represent an important source of unmeasured heterogeneity influencing both revascularisation rates and clinical outcomes. In addition, although we included studies with well‐matched groups, the possibility of residual confounding, particularly with observational data, cannot be entirely ruled out. Also, the inability to perform stratified analyses according to baseline anatomical complexity (e.g., SYNTAX score) or clinical presentation (ACS vs. CCS) represents an additional limitation. Inconsistent reporting of stratified outcome data across studies precluded pooled subgroup analysis, and potential effect modification by lesion complexity or clinical context cannot therefore be excluded. Lastly, the comparison between IVUS, an anatomical imaging tool, and FFR, a physiological assessment tool, presents conceptual limitations, as the two serve different purposes. Conclusively, although this study attempted to reflect real‐world clinical practice variations, operator expertise and accessibility of adjunctive technologies were not consistently reported and may impact study findings. Sixth, differences in institutional protocols, thresholds for PCI and access to adjunctive imaging technologies may have contributed to between‐study heterogeneity and limited the direct applicability of findings across all clinical settings. Finally, although one study applied a lower FFR threshold (< 0.75), the remaining included trials used a ≤ 0.80 cut‐off, therefore restricting formal subgroup analysis. Importantly, variability in physiological cut‐offs may influence lesion selection, as lower thresholds preferentially identify more haemodynamically severe stenoses, whereas a ≤ 0.80 threshold encompasses a broader spectrum of intermediate lesions. Such differences may affect baseline risk profiles and event rates, potentially influencing the external applicability of pooled estimates.

## 5. Conclusion

In conclusion, PCI guided by either FFR or IVUS yields a comparable incidence of MACE and individual cardiovascular outcomes in patients with intermediate coronary lesions. Furthermore, there was no significant difference in the overall number of PCI procedures between the two groups, although the evidence remains limited and heterogeneous. These findings suggest that FFR and IVUS provide equivalent long‐term outcomes, with no clear evidence to support the preference of one strategy over the other. Further well‐designed multicentre RCTs with larger sample sizes and long‐term follow‐up are warranted to confirm these results.

### 5.1. Registration and Protocol

This systematic review was registered in the PROSPERO International Register of Systematic reviews (Registration Number CRD420251082581). The protocol is available from https://www.crd.york.ac.uk/PROSPERO/view/CRD420251082581.

**Nomenclature**
FFR: fractional flow reserve
IVUS: intravascular ultrasound
PCI: percutaneous coronary intervention
MACE: major adverse cardiovascular events
RCT: randomised controlled trial
OR: odds ratio
CI: confidence interval

## Author Contributions

Eyad Jamileh collected and analysed data, contributed to drafting and revised the article. Zuha Akhtar, Kanita Farooq and Maria Babu collected and analysed data and contributed to drafting. Mohamed Xamza revised the article. Ibrahim Antoun contributed to the design of the study.

## Funding

No funding was received for this manuscript.

## Disclosure

Mohamed Xamza and Ibrahim Antoun contributed to the final approval of the manuscript.

## Ethics Statement

A statement of ethics is not applicable because this study is based exclusively on published literature.

## Conflicts of Interest

The authors declare no conflicts of interest.

## General Statement

*Impact on Daily Practice.* The findings of this meta‐analysis provide important insights into optimising PCI guidance strategies in clinical practice. Although both FFR and IVUS improve outcomes compared with angiography alone, FFR minimises unnecessary stenting by offering physiological assessment, whereas IVUS ensures optimal stent deployment through anatomical visualisation. Understanding the complementary roles of these modalities supports individualised, cost‐effective decision‐making, particularly in intermediate lesions or complex anatomies, ultimately enhancing patient safety and procedural success.

## Supporting Information

Additional supporting information can be found online in the Supporting Information section.

## Supporting information


**Supporting Information 1** The supporting information accompanying this manuscript include Table S1, which details the comprehensive search strategy used across PubMed, EMBASE, CINAHL, Web of Science and the Cochrane CENTRAL databases, and

**Supporting Information 2** File S1, which contains the PRISMA 2020 Checklist documenting adherence to the Preferred Reporting Items for Systematic Reviews and Meta‐Analyses guidelines.

## Data Availability

All data generated or analysed during this study are included in this article. Further enquiries can be directed to the corresponding author.
