# Supplementary material for: Comparative Outcomes of Fractional Flow Reserve and Intravascular Ultrasound Guidance for Percutaneous Coronary Intervention in Intermediate Lesions: A Systematic Review and Meta‐Analysis
Source: Cardiovasc Ther. 2026 Mar 27;2026:6570642. doi: 10.1155/cdr/6570642 (PMC13140804; doi:10.1155/cdr/6570642)
Supplement: Supplementary file 1 — Supporting Information 1 The supporting information accompanying this manuscript include Table S1, which details the comprehensive search strategy used across PubMed, EMBASE, CINAHL, Web of Science and the Cochrane CENTRAL databases, and [file CDR-2026-6570642-s001.docx]

## Database: MEDLINE (via PubMed):

(("Fractional Flow Reserve"[Mesh] OR "fractional flow reserve" OR FFR)
AND
("Intravascular Ultrasound"[Mesh] OR "intravascular ultrasound" OR IVUS OR "intravascular imaging" OR "ultrasonography, intravascular")
AND
("Percutaneous Coronary Intervention"[Mesh] OR "percutaneous coronary intervention" OR PCI OR angioplasty OR "stent" OR "coronary intervention" OR "drug-eluting stent" OR DES)
AND
("Major Adverse Cardiac Event*" OR MACE OR "mortality" OR "death" OR "target vessel revascularization" OR TVR OR "target lesion revascularization" OR TLR OR "myocardial infarction" OR MI OR "stent thrombosis"))

## Database: EMBASE

('fractional flow reserve'/exp OR 'fractional flow reserve' OR FFR)
AND
('intravascular ultrasound'/exp OR 'intravascular ultrasound' OR IVUS OR 'intravascular imaging')
AND
('percutaneous coronary intervention'/exp OR 'percutaneous coronary intervention' OR PCI OR angioplasty OR 'stent' OR 'drug eluting stent')
AND
('major adverse cardiac event'/exp OR MACE OR 'mortality' OR 'myocardial infarction' OR 'target vessel revascularization' OR 'stent thrombosis')

## Database: CINAHL

((MH "Fractional Flow Reserve") OR "fractional flow reserve" OR FFR)
AND
((MH "Intravascular Ultrasonography") OR "intravascular ultrasound" OR IVUS OR "intravascular imaging")
AND
((MH "Percutaneous Coronary Intervention") OR "percutaneous coronary intervention" OR PCI OR angioplasty OR stent)
AND
(MACE OR "major adverse cardiac event*" OR mortality OR "myocardial infarction" OR "stent thrombosis")

## Database: Web of Science

TS=("fractional flow reserve" OR FFR) AND TS=("intravascular ultrasound" OR IVUS OR "intravascular imaging") AND TS=("percutaneous coronary intervention" OR PCI OR angioplasty OR stent) AND TS=("major adverse cardiac event*" OR MACE OR mortality OR "myocardial infarction" OR "stent thrombosis")

## Database: Cochrane CENTRAL

("fractional flow reserve" OR FFR) AND ("intravascular ultrasound" OR IVUS) AND ("percutaneous coronary intervention" OR PCI OR stent) AND (MACE OR "major adverse cardiac event*" OR mortality OR "myocardial infarction")
